# Iodide of Potash

**Published:** 1891-07-18

**Authors:** 


### IODIDE OF POTASH.

The action of iodide of potash in removing neoplasms and in promoting expectoration during the course of certain kinds of bronchitis, as well as its action on aneurysms, and, indeed, its powerful influence on special forms of ulcers, have received some elucidation from the recent observations of Sticker. He considers that it has the same effects on the tissues as tuberculin, though in a minor degree; that it causes a more or less localised inflammation, especially in the presence of tubercular matter, which is thus isolated, destroyed, and cast out. Now, the means by which iodide of potash produces its beneficial effects have been satisfactorily explained in but few instances. Its value in lead-poisoning can be shown to depend on the formation of certain soluble double sulphates, which easily pass out of the system, but in its other applications we find no such easy and simple explanation. In many cases its value has been supposed to arise solely from its power of setting free previously administered mercury which was lying in the tissues, but however obtained, its efficiency has stood the trial of time in a manner that few other drugs have done. From the doses of a single grain which were employed when iodide was first known, we have come to see half a drachm three or four times a-day in certain cases administered with astonishingly good results. In other diseases minimum doses seem just as efficient for the purpose aimed at, and perhaps even more active in producing symptoms of what are called iodism. This curious action forms one of the greatest drawbacks to the administration of the drug; for it is perfectly impossible when administering small doses to discover beforehand those comparatively rare individuals who will all at once be found streaming from eyes and nose and mouth, and complaining bitterly of the pain, smarting, and violent headache. We neither know how these symptoms are produced, nor to what idiosyncracy they are due. The iodide is rapidly eliminated, but traces remain behind for a long while, and there seems no proof of the assertion that failure to eliminate is followed by iodism. The cause, again, cannot be an accumulation of iodide, for it occurs most frequently when small doses are given. Nor does it appear to consist in a liberation of iodine, though this may exceptionally occur just as the formation of biniodide of mercury has been noticed with serious results when mercurial ointments have been applied to the eyes after a course of iodide. In fact, we are quite unable to explain the phœnomenon, or to foresee its occurrence. Yet it is not altogether distinct from the free expectoration obtained generally by the administration of small doses in bronchitis. Now, Sticker has remarked that the usual effect of iodide given to healthy persons is nil, but if dry bronchitis exists moist rales are soon heard over the lungs. If given where there is a doubtful patch at the apex or lower down without rales or bronchial breathing, or where there is reason to think that early phthisis exists without physical signs, the uncertainty can be cleared up by a few doses of iodide, for abnormal respiratory murmurs and vales will temporarily appear. In the same way adhesions and other changes can be discovered. He insists that the tissue around a tubercular nodule is thrown into a state of activity by iodide, as it is by an injection of tuberculin; that this tends likewise to isolate and destroy the diseased tissue, and he even employs tuberculin at longer intervals than usual, and keeps up a mild reaction between the doses by administering iodide. The latter has, therefore, both a remedial and diagnostic value. He does not, indeed, suggest that the appearance of iodism indicates tubercular lesions widely scattered, but in so far as his results are held to be proved we may argue that one result of iodide administration is the production of a remedial but inflammatory activity in the neighbourhood of diseased tissues. The analogy of gummata with tubercular nodules is close and has long been recognised. The iodide which causes them to melt away so rapidly probably acts on them as tuberculin does on the tuberculous mass by irritating the surrounding cells to abnormal activity. In neither case is the disease necessarily eradicated, but the local manifestation is removed, and the spread of the virus checked in some degree by the increased activity of the tissues. Iodide would seem to have the same effect on either disease through its stimulation of the cells of the body, and the rise of temperature supposed peculiar to tuberculin may depend on the nature of the disease for which it is employed. The action of iodide in promoting the formation of clot in aneurysms is of interest here if we recall the assertion made same time ago as to the tubercular origin of these lesions. The demonstration of bacilli in the aneurysmal coats is not an easy matter, and the rarity of the coincidence of the disease with other tuberculous lesions incline us to demand more exhaustive enquiries. If, however, we can assert a tubercular nature, the efficacy of iodide becomes clear. The tissues of the sac are probably stimulated, and the resulting coagulum is the true analogue of the caseous masses produced by Koch's treatment of phthisis. We shall await with great interest the further reports of Dr. Sticker, for his late communication to the Centralblatt für Clin. Med. opens the way to a more intelligible explanation of two remedies hitherto little understood. The diagnostic value alone of iodide in early phthisis must be considerable if, as he claims, sputa containing bacilli have been thrown off by its use long before there was any other reasonable ground for diagnosis. A method of attacking and expelling phthisical foci at their very earliest formation is of the highest importance for practical medicine, and we may here find a use for iodide of potash in no degree inferior to its older applications.

